# High fidelity visualization of multiscale dynamics of laser-induced bubbles in liquids containing gold nanoparticles

**DOI:** 10.1038/s41598-018-27663-z

**Published:** 2018-06-25

**Authors:** Manoj K. Bhuyan, Antonin Soleilhac, Madhura Somayaji, Tatiana E. Itina, Rodolphe Antoine, Razvan Stoian

**Affiliations:** 1 Laboratoire Hubert Curien, UMR 5516 CNRS, Université de Lyon, Université Jean Monnet, 42000 St. Etienne, France; 2 Institut Lumiére Matiére, UMR5306 CNRS, Université de Lyon, Université Claude Bernard Lyon 1, 69100 Villeurbanne, France

## Abstract

Cavitation in pure liquids and in liquids containing nanoparticles enables applications in mechanics, bio-medicine, and energy. Its evolution carries a significant interest. We describe the multiscale dynamic evolution of ultrafast-laser-induced cavitation in pure and gold-nanoparticles-doped liquids in one-dimensional geometries induced by non-diffractive ultrashort Bessel-Gauss laser beams. Covering the complete electronic and thermomechanical cycle, from the early plasma phase to bubble cavitation and collapse on ms timescales, we reconstitute, using time-resolved imaging with amplitude and phase sensitivity, the hydrodynamic phenomena concurring to bubble evolution. We indicate geometry-specific instabilities accompanying the collapse. The insertion of gold nanoparticles of 200 nm size has subtle effects in the process energetics. Albeit a moderate field enhancement minimizing the contribution to breakdown, the nanoparticles play a role in the overall relaxation dynamics of bubbles. The evolving bubble border in nanoparticles-containing liquids create a snow-plough effect that sweeps the nanoparticles at the gas liquid interface. This indicates that during the macroscopic cavity development, the nanoparticles were removed from the interaction region and dragged by the hydrodynamic movement. We thus shed light on the evolution of cavitation bubbles not triggered but perturbed by the presence of nanoparticles.

## Introduction

The interaction of ultrashort laser pulses with liquids in energetic ranges capable of inducing catastrophic optical breakdown and subsequent cavitation carries a paramount interest^1,2^. Fluids are a topic of continuous study^3^ and this pertains not only to the fundamental perspective of understanding carrier transport in liquids, photo-disruption, cavitation, and rupture phenomena in soft matter^4,5^, but also outlines the remarkable potential in a large range of applications^6^. If the original concern was motivated by mechanical and erosion phenomena at solid-liquid interfaces originating from cavity dynamics^7^, a novel interest has appeard in medicine and biology, particularly in ophthalmology and living tissue surgery^8–11^. This interest is also fueled by the possibility to link nanoscales with fluid properties, with remarkable capacities to localize light^12,13^. With this set of applications the requirements of energy localization and controllability became inherent and of paramount importance. Spatio-temporal engineering of irradiation can maximize the impact of laser energy, augmenting the conversion rate to mechanical energy^14^. It equally allows to explore nonlinearities in propagation and ionization, reaching extreme spatial scales for energy confinement. The challenge of bypassing the diffraction limit enabled the emergence of new concepts and interaction schemes based on nanoparticles and plasmonic resonances. The insertion of nanoparticles in liquids created the possibility of localizing energy on single sites using nanometer-scale field enhancement determined by the shape and by the nature of the inserted particles^15,16^, and equally of initiating spatially selective energy transport from the laser radiation to the host. The capacity of controlling and localizing bubble cavitation enables innovative developments in nano-surgery, tissue cutting, and tumor therapy. Heat transport is equally of interest^17^. Several studies have indicated that a small amount (1% by volume) of nanoparticles can significantly change the thermal properties of fluids. Gold nanoparticles (GNPs) are being increasingly used due to their excellent thermal, optical, chemical, and biological properties^18^. Both experimental and theoretical approaches were employed to study nanoparticle-related heat transfer phenomena^19^. For laser heating of GNPs suspended in an aqueous medium, optical energy is rapidly converted to thermal energy, raising the particle temperature through a surface plasmon resonance (SPR), with subsequently transferring heat to the surrounding environment. During this process, the GNPs and the surrounding medium experience a number of various thermophysical processes, such as thermal expansion of particles, particle melting, and vapor bubble formation (plasmonic bubble PB). It was indicated^15,20^ that the size and the shape of GNPs have a great importance in determining photo-thermal effects and in the formation of bubbles; their capacity to localize or delocalize energy being important in igniting cavitation below its intrinsic threshold. A specific interest appears in the situation where the nanoparticle sizes are tuned out from the optical resonance, permitting to pinpoint their mechanical contribution. Equally, more energetic interactions and laser-induced Coulomb explosion of gold nanoparticles into tumor with the formation of cavitation bubbles has been proposed as an effective approach for selective damage of breast cancer with gold nanoparticles^21,22^.

The interest in liquids with inserted nano-objects joins also the field of interaction of short-pulse lasers with atomic and molecular aggregates for the development of secondary sources; from X-rays to neutrons or charged ions, culminating with nuclear reactions from nanosized objects^23^. Novel perspectives are thus opened in the field of energy harvesting where concepts of particle fission and neutron generation are now emerging^24^. Engineered nanoparticles are considered potential sources for facilitating proton induced reactions, releasing energy as heat and avoiding generation of nuclear byproducts such as neutrons^25^. Considering heat collection and confinement conditions, liquids are regarded as interesting options for collecting excess energy in a nuclear environment. This motivates therefore the study of cavitation effects in liquids containing nanoparticles, notably the interaction of the nanoparticles with laser-induced bubbles. Here an important aspect concerns the hydrodynamic motion of the liquid containing the particles. It is of a significant importance for the field of energy harvesting to investigate the role of shock waves and bubble formation, and to check if particles will stay localized or will move with the liquid after laser excitation.

The hydrodynamical processes involved in laser-induced cavitation and bubble formation in liquids are fairly known, from the bubble expansion to its collapse and the formation of inner and outer jets. Water is the medium of choice in a large majority of studies. The typical scenario involves the creation of a charge carrier plasma, usually assumed at the critical density for the corresponding irradiation wavelength, initiating an optical runaway, or at sub-critical densities at the cavitation threshold. Electronic relaxation is followed by electron vibrational interaction and by the rise of the liquid matrix temperature and pressure on a ps timescale. The mechanical relaxation via pressure waves triggers tensile stresses and initiates the cavitation of the liquid and the onset of a bubble. Several studies focused on the excitation parameters in relation to cavitation threshold^5,26^ or followed nonlinear propagation of ultrashort laser pulses in water^27^, with specific works dedicated to dynamic techniques for monitoring cavity development^28,29^. More recent, the interaction of non-standard beams received increased attention due to particular field distributions. This is the case of the non-diffractive zero-order Bessel beams^30^. The cavitation geometry acquires a one-dimensional character^31,32^, with the subsequent distribution of radial pressure. Applications in biology were indicated^33^, increasing the flexibility in dissecting subcellular domains.

In this paper, we evaluate the possible influence of the presence of gold NPs on the bubble hydrodynamics, for a cavitation geometry of particular form having a cylindrical evolution symmetry. Ultrashort non-diffractive Bessel beams were used to trigger cavitation by creating a uniform excitation domain of high aspect ratio. Then high-resolution optical imaging synchronized with the characteristic times of the event is used to visualize the phenomenon. The bubble complex evolution is observed using a high fidelity *in-situ* time-resolved optical monitoring technique based on dynamic optical transmission and phase contrast microscopy, where the degree of coherence of the illumination can determine an outstanding level of observing details and particles effects. In order to have benchmarks, we first discuss the multiscale dynamics of laser-induced cavitation in water, water-solvent mixtures and solvent (ethylene-glycol) in non-diffractive irradiation modes following excitation and relaxation ranges. Using time-resolved imaging with amplitude and phase sensitivity on multiple (fs to ms) timescales, we reconstitute with high fidelity the hydrodynamic phenomena leading to bubble evolution as well as the instabilities that occur inside bubbles generated in one-dimensional geometries around the point of collapse. This gives a perspective on the complete evolution of the irradiated liquid domain, from the early plasma excitation phase in the first fs to liquid cavitation following pressure relaxation and subsequent bubble collapse on *μ*s timescales. Information related to excitation degree and the evolution of the cavity interfaces are obtained from analyzing both transmission and associated optical retardance. The second question we tried to answer is related to irradiation of liquids containing nanoparticles. Following the interest in the interaction of ultrashort ultra-intense pulses with aggregates in particular environments, we interrogate the ability of the nanoparticles to survive primary excitation and resist within the bubble. In particular, we will analyze the interface capability to sweep and remove nanoparticles during the bubble expansion. The evolving bubble border in nanoparticles-containing liquids creates a snow-plough effect that traps and sweeps the nanoparticles at the gas liquid interface. This observation indicates that during the macroscopic cavity development, the nanoparticles were removed from the interaction region and dragged by the hydrodynamic movement. Trapping of nanoparticles in a liquid by laser-induced microbubbles has already been reported^21^. However, another technique was used to capture and manipulate nanoparticles, based on optically-induced thermophoresis and convection phenomena^34^. Convective flow can exert a force on the particle^35^ at a relatively far distance from the bubble (about 500 *μ*m) with a velocity of 90 *μ*m/s^−1^. Clearly such “slow” phenomenon cannot explain the snow-plough effect, since nanoparticle trapping occur during bubble expansion within few microseconds. In the present “snow-plough” effect, when the nanoparticle is brought into contact with the bubble, the surface tension and pressure force dominate and the balance between these two forces traps the particle against the bubble surface. For this purpose, the evolution of the irradiated liquid domain with and without GNPs is compared in the expansion and collapse phase. The manuscript is organized in several parts. The experimental method is first presented showing the irradiation geometry and the two time-resolved approaches on multiple timescales. The ultrafast sub-ps and ps dynamics is first discussed. Then the slow thermomechanical evolution is indicated over the whole evolution cycle. Considerations on the cavitation thresholds are given. Thirdly, the presence of gold nanoparticles is analyzed for a possible influence on the bubble hydrodynamics and for interrogating their capacity of surviving inside the bubble after the passage of the laser pulse. The sweep-out role of the bubble expanding interface is outlined.

## Methods

The section includes a description of the excitation geometry, of the sample preparation and of the observation techniques.

### Experimental procedure: Non-diffractive beams

The laser source consists of an amplified ultrafast Ti:Sapphire system. The system delivers 60 fs laser pulses at 800 nm central wavelength and at 1 kHz repetition rate. The laser pulse duration can be tuned in the fs-ps range using additional second-order dispersion by adjusting the compressor inter-grating distance. High contrast single pulses can be extracted using opto-electronic and mechanical shutters. Several methods exists for generating non-diffractive beams by conical intersection (see ref.^36^ and references therein). We opted here for axicon generated Bessel-Gauss beams (referred hereafter as Bessel beams). Zeroth-order Bessel beams^30^ are generated using an axicon lens with an apex angle of 179°. The conical phase pattern of the axicon bends the incoming light to the axis. It thus determines a transverse to axial light transformation, where the incoming wavefronts create a stationary field encompassing a narrow intense central core axially elongated over a large distance (as compared to the Rayleigh range of an equivalent Gauss beam) and surrounded by low intensity rings following the Bessel transverse profile. The central core and the rings are the consequence of successive constructive interference relations of the incoming conical wavefront. The Bessel beam is then demagnified and imaged inside a water cuvette using a 4 f afocal imaging system^37^ with a microscope objective as the end optical imaging/focusing element. Bessel beams with different cone angles can be obtained by employing objectives with various optical powers. Mostly moderate and tight focusing conditions (objective numerical aperture of NA = 0.28 and 0.42) are used inside the liquid, with half-cone angles *θ* = 9° and 16.5° resulting in FWHM core diameters of 1.5 and 0.7 *μ*m respectively. The aspect ratio lies around 100. For observing the laser-excited regions in liquids, *in-situ* and in real-time, dynamic optical transmission (OTM) and positive phase-contrast (PCM) microscopy are employed. Several water (distilled) and water-ethylene glycol mixtures were used and in particular cases they were doped with gold nanoparticles.

### Experimental procedure: Nanoparticles and solvent

Gold nanoparticles of 200 nm size (hydrodynamic radius 175–235 nm, polydispersion index 0.3) stabilized in suspension in a citrate solution (742066–Sigma Aldrich) were chosen as their absorption cross-section at the spectral wavelength of 800 nm shows a minimum, with diffusion efficiency achieving a maximum value. This choice is equally motivated by the possibility of detection by optical means while avoiding sedimentation issues at larger dimensions. The particles are dispersed in a solution of water and ethylene-glycol (70:30%).

### Experimental procedure: Time-resolved microscopy

The complete relaxation dynamics following ultrashort pulse excitation of liquids in non-diffractive tight focusing conditions is observed using multiscale time-resolved imaging in a microscopy arrangement. The laser-excited liquid evolution is followed for time domains starting with the ultrafast excitation and extending to relatively slow cavity hydrodynamics down to final relaxation. The method allows for the observation of the laser-affected region in phase and amplitude (delivering information on absorbance and phase shifts) for a timescale ranging from fs to ms and with sub-micron spatial resolution. It consists of two experimental arrangements, one allowing for inspecting the ultrafast dynamics (UFD) below ns with sub-ps resolution, the second multiscale (MSD) one being optimized for long-range mechanical relaxation of the liquid, up to the ms. The imaging setup is based on an upright optical microscope (Olympus BX-51) operating in optical transmission (OTM) and phase-contrast (PCM) diascopy modes. In OTM, absorbing or scattering regions appear dark on a bright background. In PCM, phase shifts corresponding to negative and positive refractive index changes appear bright, respectively dark on a grey background, provided that the phase shift stays comparable to the observation wavelength. It has to be noted that the relative index and phase changes observed in PCM represent qualitative variations of the initial quantities. For each time domain sources with various degrees of spatial coherence were used.

#### Ultrafast dynamics; the fs-ps evolution range

The first domain of interest relates to fs-ps-ns evolution. The primary excitation dynamics and relaxation channels in ultrafast domains associated with laser-liquid interaction are observed with time and space resolution using a standard two-color pump-probe time-resolved microscopy technique. This delivers instantaneous excitation charts at various time moments during and after excitation and covers a time domain from fs to ns. A strobe light illumination method time-synchronized with the exciting beam is used, employing 400 nm ultrashort probe wavelengths. This allows to avoid coherent artifacts between pump and probe pulses and enables better separation. The probe pulses were formed by sampling a part of the main beam and injecting it in an optical delay line, ensuring thus precise optical synchronization. The 400 nm probe photons were obtained by frequency doubling the input 800 nm beam in a thin BBO crystal. Pulse duration can be varied in fs and ps ranges. The pump and the probe have the same temporal duration, however, since fs and ps pulse will respectively probe dynamics much longer than the probe duration, no loss on measurement precision is identified at passing from fs to ps ranges. The excitation region is imaged from an orthogonal direction using an approximate Köhler illumination technique in transmission, with limitations due to the cuvette dimensions. This geometry provides information on the amplitude and the phase of the illuminated object in ultrafast OTM_*UFD*_ and PCM_*UFD*_ modes with a view sectioning the image in planes parallel to the propagation axis along an orthogonal path. The probe enters the illumination path of the microscope via the microscope condenser and the optical transmission and phase-contrast microscope images are recorded in the image plane with a back-illuminated electron multiplier EMCCD (ANDOR iXon Ultra 897) camera. Mostly an objective of NA = 0.4 was used for observation, giving a resolution of 600 nm. A 10 nm bandwidth pass filter centered at the probing wavelength is used to cut the parasitic incoherent light emitted by the excited region and the scattering from the pump pulse. The temporal delay of the probe pulse with respect to the pump is varied using a motorized translation stage. The ultrashort illumination source is a spatially coherent source and source of speckles. In view of its short duration, the degree of coherence cannot be easily altered during the pulse. Rotating diffuser plates are used to reduce the average spatial coherence of the probe for multipulse exposures, increasing the uniformity of illumination. Snapshots are averaged over 40 images where the sample is moved to a new position between each image. A detailed description of the setup is given in ref.^38^.

#### Multiscale (ns-ms) relaxation and cavitation

The long range relaxation dynamics following ultrashort pulse excitation of the liquid in non-diffractive tight focusing conditions is observed using multiscale time-resolved imaging. This corresponds to a comparatively slow hydrodynamic evolution of the bubble. The method allows for the observation of the laser-induced evolving bubble in phase and amplitude for a timescale ranging from ns to ms, and with sub-micron spatial resolution, covering the whole bubble dynamics. A similar two-color pump-probe microscopy technique with respect to the ultrafast dynamics part is used, equally responsive to relative differences in the object optical phase and amplitude characteristics, and having this time ns temporal resolution. This collects corresponding charts of material transformation at various illumination time moments after excitation where amplitude and phase contrast allows to enhance the observation accuracy. The pump pulse is equally represented by an 800 nm ultrashort laser pulse of fs or ps duration, shaped in non-diffractive mode. For the microscopy part, the observation objective of NA = 0.55 gives a spatial resolution of 650 nm at the observation wavelength (590 nm). The stroboscopic illumination method is used as mentioned above, where the probe pulse cross-illuminates the interaction zone in an orthogonal geometry. The probe pulse is generated this time from an independent light source. To obtain high-resolution single-shot images with uniform background and low speckle noise, a low spatial coherence pulsed source is developed based on a random lasing effect^39^, as described in ref.^40^, by creating a disordered gain medium. Thus, the laser gain medium consists of a colloidal solution of Rhodamine B (2.5 g/l) with immersed latex nanobeads of 325 nm size. The concentration of latex particles is 4 × 10^15^ l^−1^. The random lasing effect is obtained by pumping the colloidal solution with 532 nm laser pulses in the absorption band of the solution. Stimulated emission occurs where multiple scattering creates the random character. The exciting laser pulses in the solution were obtained from a frequency doubled Nd:YAG laser operating at 10 Hz and delivering 7 ns pulses in the mJ energy range. The random lasing effect is detected via the appearance of a strong spectral narrowing of the otherwise large red fluorescence band to around 13 nm bandwidth (FWHM) centered at 590 nm. The random laser pulse duration is similar to the excitation pulse in solution, i.e. 7 ns. The bright low-coherence narrow-band illumination source has the potential of acquiring high quality speckle-free images^41^, providing at the same time information on the amplitude and on the phase of the object. This illumination method increases significantly the dynamical range of the image and the signal-to-noise ratio, allowing to take full advantage of the optical resolution of the microscope with time resolution. The electronic synchronization between the ultrafast laser system and the ns laser system ensures the time-synchronization between the exciting ultrashort laser pulse in liquids and the random lasing illumination (probe) source. The typical jitter is few tens of ns and for small delay times it was measured with the help of a fast photodiode. Similar to the ultrafast method, the excitation region in the liquid sample is imaged in a perpendicular geometry using an arrangement close to Köhler illumination geometry, with limitations due to the cuvette dimensions. The probe enters the illumination path of the microscope via the microscope condenser and the optical transmission (OTM_*MSD*_) and phase-contrast (PCM_*MSD*_) microscope images are recorded in the image plane with the back-illuminated electron multiplier EMCCD. A 20 nm bandwidth pass filter centered at the probing laser wavelength (590 nm) is used to cut parasitic incoherent light emitted by the specimen and the scattering from the pump. The characteristics of the method are further illustrated in ref.^42^.

## Results and Discussion

### Ultrafast dynamics of bubbles in pure liquids

**Figure 1:**
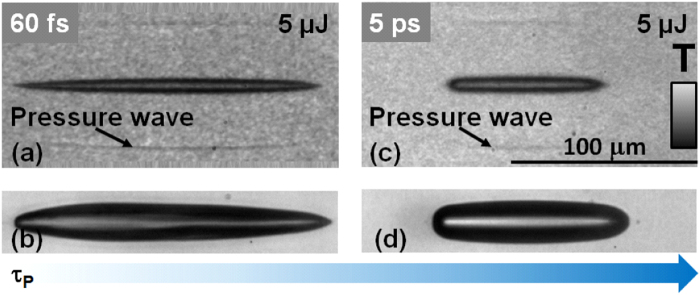
Bubble cavitation in water. Snapshots of initial stages in bubble formation and development using ultrashort (fs) and short (ps) Bessel pulses, in tight focusing configuration (*θ* = 16.5°), observed in transverse OTM. (**a**,**b**) The cavity development for the ultrashort pulse case. (**a**) OTM_*UFD*_ cavitation snapshots observed at 17.5 ns time delay showing the typical stability of cavity development for the ultrashort pulse case (60 fs). A short 400 nm probe was used. The input energy is 5 *μ*J, above the cavitation threshold (*E*_*th*1_ = 0.6 *μ*J). (**b**) The cavity aspect at 100 ns time delay for the respective pulse duration and energy (OTM_*MSD*_ mode). (**c**) The cavity development for the short pulse case (5 ps) in OTM_*UFD*_. The input energy is 5 *μ*J, above the cavitation threshold at this pulse duration (*E*_*th*2_ = 3.5 *μ*J). The ps irradiation generates more uniform cavitation events with respect to the ultrashort pulse case, due to a better regulated nonlinear interaction. (**d**) The cavity aspect at 100 ns time delay for the respective pulse duration and energy (OTM_*MSD*_ mode).

We first briefly discuss the irradiation conditions capable of generating stable, unperturbed geometries for cavitation within the liquids, reflecting the ideal geometry of the deposited energy in terms of uniformity. Typically ultrashort pulse laser propagation in a nonlinear environment is affected by nonlinear distortions. Pulse distortions are intrinsically related to the material and can be equally self-induced, depending on the local laser intensities and the capabilities of generating local plasmas. These effects include among others self-focusing, diffraction, and light defocusing. If Bessel beams are considered more nonlinearly stable with respect to the Gaussian pulsed beams, self-focusing and plasma generation are nevertheless major sources of disturbances in energy deposition^43^. In this respect we have compared fs and ps pulses for liquid excitation to retrieve optimal uniform energy deposition conditions, as pulse duration can define local intensities and set the dynamics of excitation. This is illustrated in Fig. 1 for two pulse durations in the fs and ps range in conditions of tight focusing (*θ* = 16.5°). The figure shows the laser-induced cavity in water at two specific time moments (17.5 ns in ultrafast microscopy and 100 ns in multiscale microscopy) for the particular irradiation conditions. These two moments are chosen to show the mechanical relaxation via the release of pressure waves and a characteristic moment in the cavity growth. For incident ultrashort laser pulses of 60 fs (Fig. 1(a,b)), the cavity geometry reflects a slightly-disturbed rather well-shaped Bessel-Gauss geometry. This remains valid for pulse energies up to 30 × *E*_*th*1_, where *E*_*th*1_ = 0.6 *μ*J is the cavitation threshold in the given conditions. The slight perturbation is observed in the early cavity phase at 17.5 ns delay (Fig. 1(a)) and better in the cavity development phase at 100 ns (Fig. 1(b)). The evolving cavity preserves the geometry of the source. Beyond these pulse energies, severe distortions appear which we associate with nonlinear propagation factors^32^. These distortions are visible in the onset of strong inhomogeneous features at the liquid gas interface. However, the remarkable feature is that increasing the pulse duration allows to significantly gain in beam propagation stability, with uniform cavities achievable beyond 30 × *E*_*th*2_ (*E*_*th*2_ = 3.5 *μ*J for a 5 ps pulse). The corresponding images are given in Fig. 1(c,d) in the early stage (Fig. 1(c), for a delay time of 17.5 ns) and in the expansion stage (Fig. 1(d) at 100 ns observation time). The capability of laser pulses of a longer pulse duration to stabilize dimensionally the energy deposition regions is an intrinsic characteristic of a dielectric^38,44^ and is significantly influenced by the plasma generation dynamics. The analyse of the magnitude of the pressure waves launched in the environment for various pulse energies in the cavitation range (from 6 to 60 *μ*J) for fs and ps pulse durations shows comparable levels. This can be observed in the contrast of the optical signatures (the transient phase shift). This facts indicates in this case that despite the difference in the cavitation threshold values, for energies well-exceeding the threshold, similar energy deposition levels are found for both pulse durations, a consequence of the mix of multiphoton processes and free carrier heating. A similar picture is found for moderate focusing geometries (*θ* = 9°), with an increasing bubble uniformity observed for ps pulse durations. Relying on the higher cavity uniformity achieved with ps laser pulses, we will discuss below the primary steps in the cavity formation using primarily laser pulses of ps durations, illustrating fs cases only when relevant for the cavitation dynamics.

**Figure 2:**
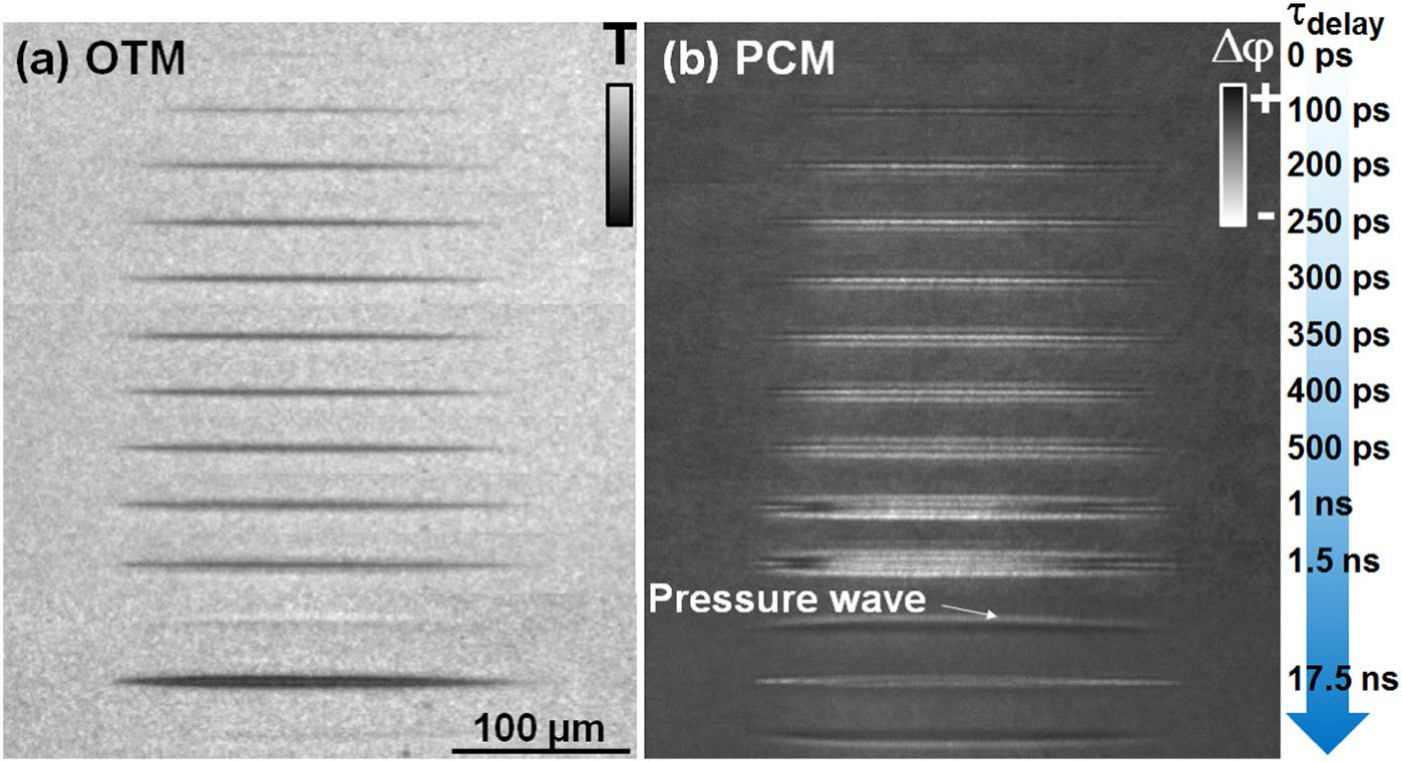
Primary cavitation stages. Ultrafast dynamics in the process of bubble formation in water in case of short (ps) Bessel pulse irradiation. A moderate focusing geometry is used (*θ* = 9°) for a 7.4 *μ*J incident pulse of 3 ps duration. (**a**) Time-resolved OTM_*UFD*_ on ps timescale, showing a gradual absorption-like response resulting from the superposition of the scattering and absorption signatures of a long-living electron plasma and the onset of cavity formation with sub-*μ*m sections. (**b**) Time-resolved PCM_*UFD*_ on ps timescale showing a gradual negative index change response resulting from the superposition of the free carriers and rarefaction signatures. The 17.5 ns observation delay time was chosen to show the development of the emerging pressure wave with an abundance of details in the PCM mode. The white domain preceding the pressure wave may be associated with a halo inherent to Zernike PCM or to a slight detuning from the Köhler illumination geometry. The slight light color preceding the pulse in OTM_*UFD*_ confirms the presence of a leading compression front.

Figure 2 shows the first stages in the cavity formation in water by a short 3 ps laser pulse in a moderate focusing geometry (*θ* = 9°). The overall cavity evolution pattern stays similar in the case of a fs laser pulse. The dynamics is now captured in OTM_*UFD*_ (Fig. 2(a)) and PCM_*UFD*_ (Fig. 2(b)) modes. The OTM_*UFD*_ shows the slow development of absorptivity. The laser excitation is signaled by the onset of a visible decrease of transmission, equivalent to an absorption signature. This is used to set the time zero of the pump-probe synchronization. We then observe that the decrease of transmissivity is gradual and peaks around 200–300 ps after the laser excitation, followed by a slow recovery. The process can be accelerated at higher energy inputs. Albeit potential changes in the carrier plasma parameters (notably in the collision time with the internal energy and thus with the delay time), this dynamics seems not to be consistent with the accepted Drude-like behavior of a carrier gas that thermalizes and cools down via vibrational activation in around 10 ps. Plasma lifetimes of several tens of ps were reported in ref.^45^ and we observed weak short plasma photoluminescence (the plasma lifetime measured with a gated ICCD is inferior or comparable to the detection ns gitter). We believe that this dynamics shows only partially the absorptive behavior of the electron-hole plasma; the overall response being superposed on the onset of a scattering element, the appearing cavitation bubble. The whole dynamics becomes then dominated by the hydrodynamic evolution of the ignited plasma, and the initiation takes place on tens to hundred ps, consistent with expansion velocities of tens to hundred m/s. At longer delay times, the emerging pressure wave becomes visible. The mechanical wave travels at supersonic speeds (a value of 1950 m/s is detected at this time moment and higher propagation speeds of several thousands of m/s occurs at lower delay times) indicating a shock-like behavior. In parallel, the PCM_*UFD*_ mode shows the negative (white) phase change corresponding to an index decrease in the excited liquid. This index decrease is potentially the combined results of the presence of light, mobile free carriers and material density rarefaction. Analyzing the OTM_*UFD*_ images close to zero delay, based on the weak transmission (few percents) we observe and, assuming standard parameters for the charge carriers (free electron mass and 1 fs collision times^8^), we deduce low electronic densities in the range of 0.1 × 10^21^ cm^−3^. These are indeed sufficient carrier densities to induce local temperature rise of the liquid matrix in the few 100 K range. To summarize this first point, the ultrafast time-resolved image series confirm thus a gradual development of transmission and phase signatures originating from the overlap of electronic excitation and mechanical cavitation in water. The latter is equally signalized at latter times via the emergence of travelling pressure waves as residuals of the pressure-induced mechanical interaction.

It is important at this point to perform a first quantitative analysis of the energy deposition. Vogel *et al*.^5^ associated the cavitation threshold with the rupture of the elastically stretched liquid due to tensile stresses related to initial pressure relaxation. The threshold initiating parameters involve local peak temperatures above 100 °C and stress values around −60 MPa, exceeding the stability limit (the spinodal limit) of water. These values of the thermodynamic parameters are achieved at rather low electronic densities, in line with the excitation dynamics observed before. We confirm these conditions by analyzing the bubble evolution up and beyond the maximal extension using Bessel beams and one-dimensional geometries.

### Multiscale dynamics over the whole cavity evolution cycle

We have observed in the ultrafast dynamics that the cavitation response overlaps with carrier relaxation. However, a relatively long electronic decay time can be detected. A mechanical response follows rapidly, with the emission of supersonic pressure waves. The cavitation process is therefore initiated by the creation of an electron-hole plasma with sufficient number density and energy density to determine bond perturbation, with relatively elevated but sub-critical carrier populations and expectable energies per electron in the ten eV range (as inferred from excitation models applied to wide band gap dielectrics^38^). This phase has therefore plasma characteristics where carrier dynamics is no longer dominated by the chemical bonding of water with ps dynamics^45^ but by charge interactions in the plasma phase close to breakdown conditions. The carrier excitation triggers local temperature and pressure increase, with a gradient that induces elastically stretching of matter. Relaxation occurs by the emission of supersonic shock-like pressure waves. Subsequent tensile stress determines the rupture of the liquid and the internal pressure drives the cavity expansion in the presence of rarefaction. Using the multiscale imaging technique we observe now the evolution and relaxation of the bubble down to its collapse and the arrest of the perturbation. The timescales that will be given below are affected by few tens of ns jitter. For large delays, the delay times are reported with an accuracy of 10%. The multiscale technique will allow for observing the cavity evolution for timescales compatible with the cavity driving factors, the tensile forces and the vapor pressure. The bubble evolution scenarios around and above the cavitation threshold and the models associated with cavitation were previously discussed (see refs^5,6^ for recent reviews). The speckle-free pulsed imaging technique offers in this context an outstanding visualisation quality of both amplitude and phase details of the evolving object.

**Figure 3:**
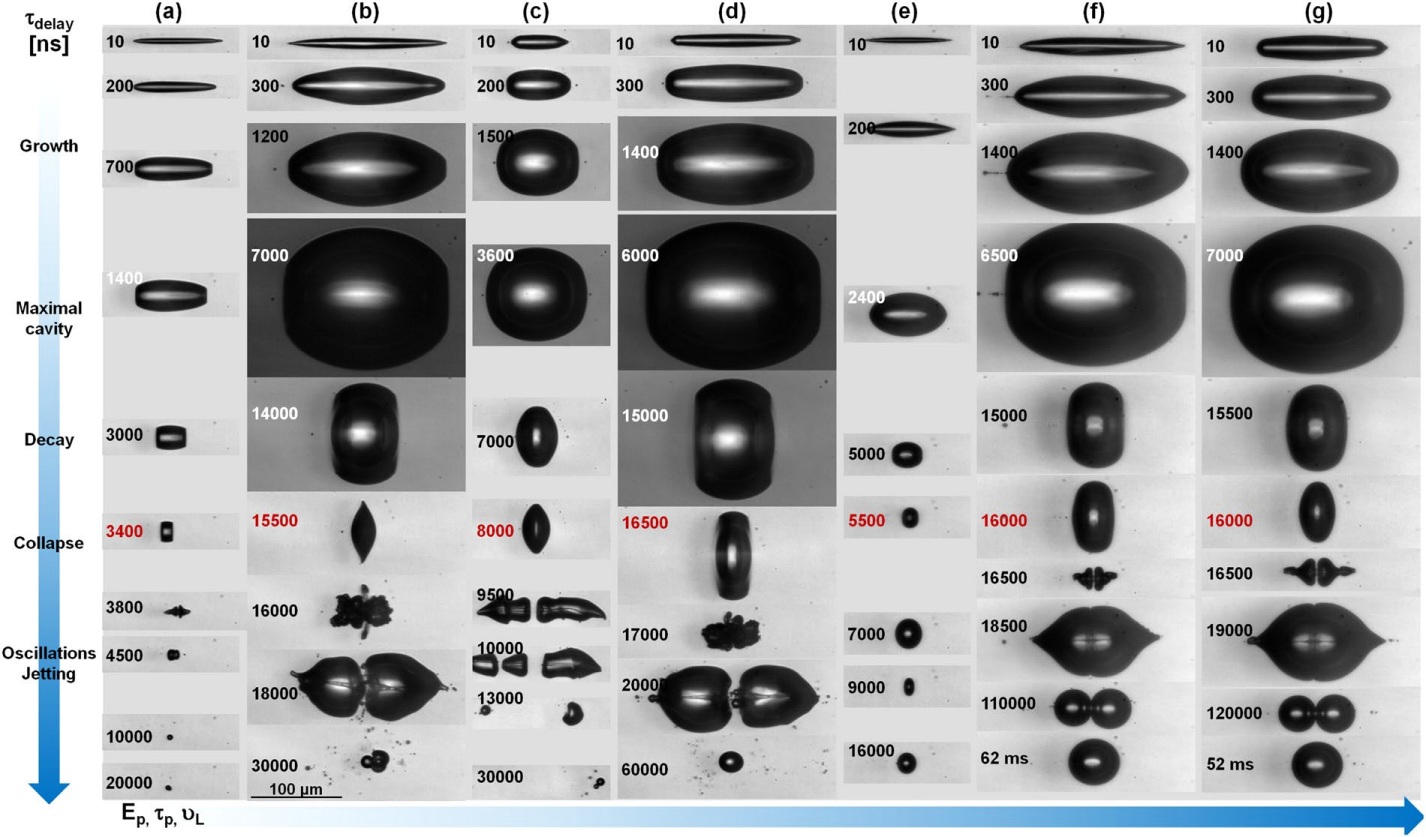
Full cycle observation. Multiscale cavitation dynamics in various liquids for different energies, pulse durations, and liquid viscosities. A tight focus configuration is employed (*θ* = 16.5°). A kaleidoscope of cavitation collapse phenomena resulting in directional jetting, bubble stabilization, or fragmentation can be obtained. (**a**) Dynamics of ultrashort pulse laser (60 fs) generated cavity in water for 1.6 × *E*_*th*1_; 1 *μ*J. (**b**) Dynamics of ultrashort pulse laser (60 fs) generated cavity in water for 9 × *E*_*th*1_; 5.4 *μ*J. (**c**) Dynamics of short pulse laser (2 ps) generated cavity in water for 1.6 × *E*_*th*3_; 2.7 *μ*J. (**d**) Dynamics of short pulse laser (2 ps) generated cavity in water for 5.2 × *E*_*th*3_; 8.8 *μ*J. (**e**) Dynamics of ultrashort pulse laser (60 fs) generated cavity in ethylene glycol for 2 × *E*_*th*5_; 1 *μ*J. (**f**) Dynamics of ultrashort pulse laser (60 fs) generated cavity in ethylene glycol for 11 × *E*_*th*5_; 5.4 *μ*J. (**g**) Dynamics of short pulse laser (2 ps) generated cavity in ethylene glycol for 4.2 × *E*_*th*6_, 5.4 *μ*J. In the two latter cases a similar value of input energy is used. The characteristic processes are indicated on the left.

An overview of the bubble cavitation dynamics in different conditions related to the input laser pulse energy, laser pulse duration, liquid viscosity is given in Fig. 3. The images show cavitation dynamics in the vicinity and well-above the cavitation threshold, emphasising particular features coming from the pulse duration and the liquid viscosity. The dynamics in pure water (viscosity 1.0 mPa s) is followed in Fig. 3(a–d). The observed evolution shows the rather restricted cavity dynamics at low energies (Fig. 3(a) for 1.6 × *E*_*th*1_), with a growth phase up to around 2 *μ*s and a collapse at around 3.6 *μ*s, followed by long living oscillations of a single bubble without fission. The growth phase follows the expected distribution of pressures in the Bessel core cylinder and the radial pressure gradient. The evolution of the cavity happens dominantly perpendicular to the laser axis (Bessel central axis), preserving the aspect ratio at low input energies. In the decaying phase towards the collapse, the velocity field changes, with maximum inverse acceleration along the axis.

The use of higher energies will trigger a longer growth phase of the cavity (Fig. 2(b–d)), higher radius, and overall longer cycles. With the increase of the deposited energy the expansion domain becomes spherical around the maximum expansion phase. The examples in the figure refer to three cases; an ultrashort (60 fs) excitation at 5.4 *μ*J, 9 × *E*_*th*1_, and a short pulse (2 ps) excitation at 1.6 × *E*_*th*3_; 2.7 *μ*J, respectively 5.2 × *E*_*th*3_; 8.8 *μ*J. We note a relatively similar evolution in the general features, with the 60 fs, 9 × *E*_*th*1_ case being quite close to the 2 ps, 5.2 × *E*_*th*3_ case, despite the difference in the absolute input energy values. This reflects a similar quantity of deposited energy *E*_*abs*_ in the two cases. For a given pulse duration, the variation of the maximal radius of the bubble is a measure of the input energy in the interrogated energy range. What is equally important to notice is that the bubble collapse in these above-threshold conditions determines a rich panorama of hydrodynamic events including spatial perturbation and modulation of the liquid-gas interface, and the onset of inner-jetting. Jetting directions will be defined by the asymmetries of the collapsing interface. The local pressure distribution plays a paramount role and may push the relaxation path either to bubble fragmentation or to a stabilization of the bubble position for relatively long times during the oscillation phase. The time-resolved technique allows capturing with high fidelity the ensemble of phenomena triggered by pressure and surface tension.

Adding ethylene-glycol to the water changes the overall viscosity and photosensitivity. The extreme case is represented by a fluid containing 100% ethylene-glycol, with the highest viscosity in the present case (viscosity of 17 mPa s). As expected for the high viscosity liquids, the cavity dynamics becomes more deterministic, particularly in the collapse phase. A first dynamic example is given in Fig. 3(e) at 2 × *E*_*th*5_, 1 *μ*J. At higher energies, we note a remarkable similarity for fs and ps excitation (Fig. 3(f,g)) for the same input energy, despite the difference in the cavitation threshold.

A bubble formation and expansion mechanism can originate from two related and even coexisting factors; the expansion of a rapidly nucleated high temperature, high-pressure gas-phase accompanied by emission of pressure waves or the relaxation of an initial impulsive high pressure phase with shock generation and the liquid rupture (spall) under tensile stress generated upon pressure unloading. While the latter (mechanical) scenario is expected around the threshold, the former is facilitated by high energy deposition rates. At typical initial electronic densities in the range of 10^20^ cm^−3^ with electronic temperatures comparable with the bandgap (the energy gain of the electrons is being limited by collisional ionization), the electronic pressure lies in the tens-hundreds MPa range. Of paramount importance is the rate of nucleation versus mechanical relaxation^46^, strongly dependent on temperature and on the possibility to achieve close-to-critical or supercritical states, i.e. explosive nucleation and fluid decomposition. Even though a Rayleigh-Plesset formalism cannot be directly applied in view of the cylindrical geometry of the cavities, the hydrodynamic behavior of the bubbles and their maximal extension is well captured by generalized Rayleigh-Plesset physical model^47^. The bubble maximal volume scales with the deposited energy, and thus the radius scales with the square root of the energy. Fitting the experimental expansion data, initial gas-liquid interface velocities are found in the rage of 100 m/s.

### Dynamics of laser-induced bubbles in liquids containing gold nanoparticles: The snow-plough effect

**Figure 4:**
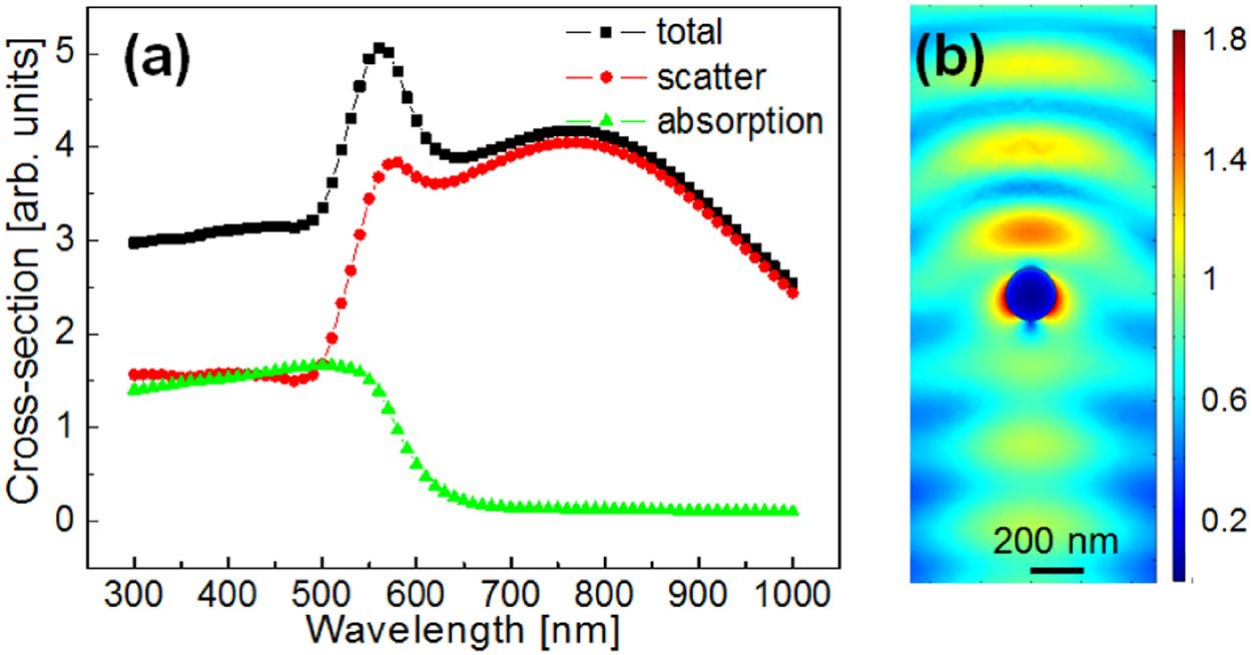
(**a**) Nanoparticles interaction with light. Total, scattering, and absorption cross-sections for a 200 nm diameter gold nanoparticle in water. (**b**) Field distribution around a 200 nm gold nanoparticle in water as a function of the light wavelength. A high scattering efficiency is expected in the present experimental conditions.

Considering these generic cavitation features, we will now compare the effect of the insertion of nanoparticles in the liquid. The objective is to test the particles capacity of existing in conditions of cavitation, as well as their role in the process itself. We will therefore make a parallel between the evolution of the laser-induced bubble in a water and water ethylene glycol mixture (70:30%) containing gold nanoparticles at a concentration of 1.3 × 10^12^ l^−1^. Particles with 200 nm diameter were chosen based on their light scattering and absorption rates. The reason for the choice lies in their scattering cross-section at 800 nm incident light which surpasses the absorption cross-section. An overview of the cross-sections for a 200 nm gold nanoparticle in water is given in Fig. 4(a). Therefore a main effect lies in the scattering of laser radiation rather than in the creation of localized centers of energy deposition. Secondly, the field enhancement around the nanoparticles stays moderate (Fig. 4(b)) minimizing their contribution to the optical breakdown of the surrounding liquid medium. The figure shows the distribution of the electric field around the nanoparticle, calculated using a 2D finite-difference time-domain (FDTD) approach.

**Figure 5:**
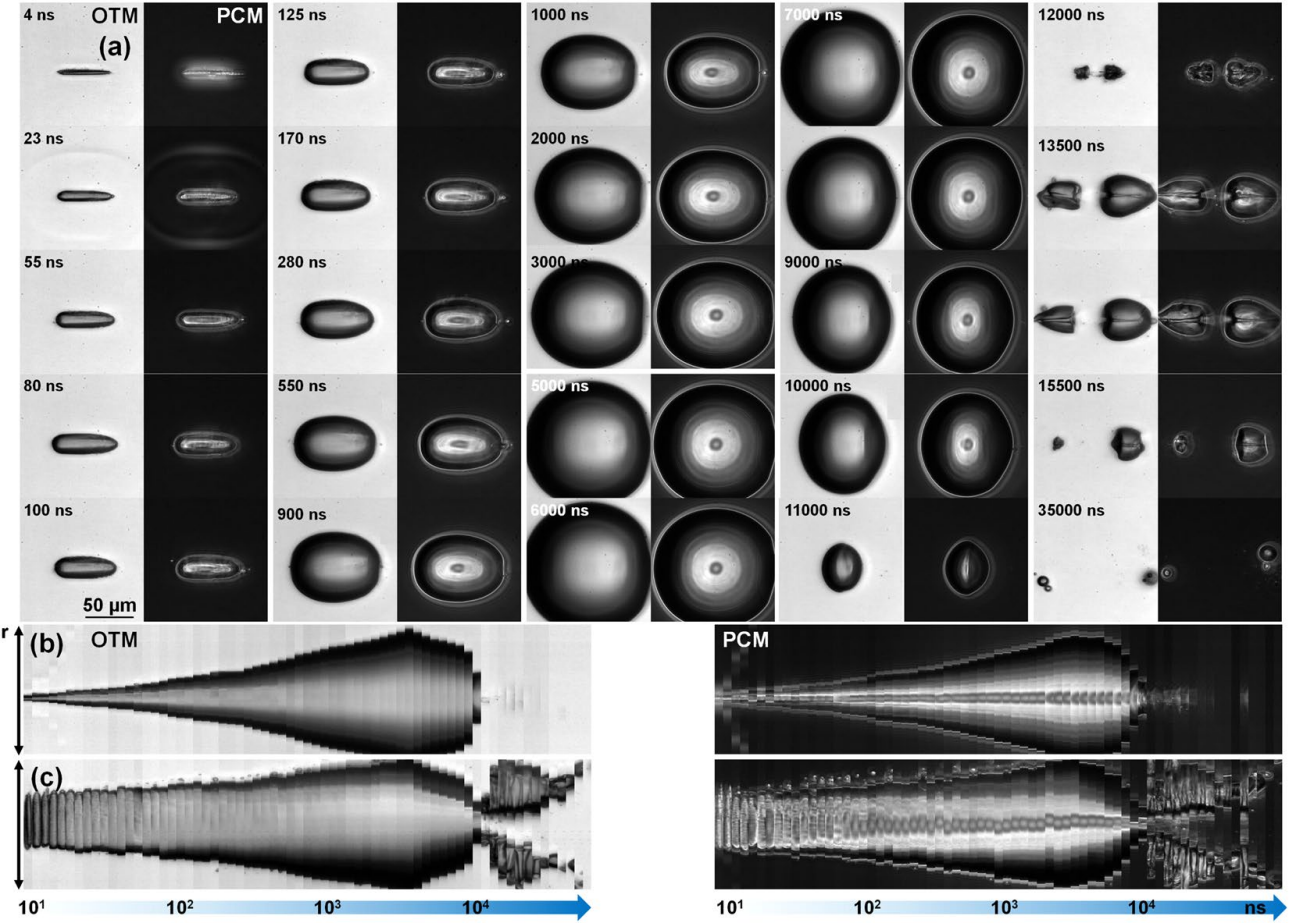
(**a**) Laser-induced cavitation dynamics in water for a tightly focused Bessel beam (*θ* = 16.5°) of 5 ps pulse duration and 4.5 *μ*J input energy. The dynamics is observed in OTM_*MSD*_ and PCM_*MSD*_ modes as indicated on the figure. Note that alternate columns represent OTM and PCM images respectively. (**b**) Dimensional dynamics of cavity evolution along the vertical axis. (**c**) Dimensional dynamics of cavity evolution along the horizontal axis, the longitudinal propagation axis. A line section was vertically, respectively horizontally made through the bubble center at each delay time and the results were concatenated to form the image. The time was plotted on a logarithmic scale. The time labels are given with a tolerance of 10%.

Let us now follow closely the cavitation dynamics in the liquids. Figure 5(a) follows the evolution of the expanding bubble in reference water using transmission and phase contrast microscopy, where the combination of the two techniques allows for complementarity in observation. The irradiation conditions involve tight focusing (*θ* = 16.5°), 5 ps pulse duration and 4.5 *μ*J input energy. The dual mode dynamic imaging allows to observe to a great detail and with high fidelity the expansion of the bubble, the liquid-gas interface movement, the collapse, and the fragmentation in the aftermath. Further analysis of the bubble movement is given in Fig. 5(b,c). These parts depict the time evolution of a line section made either vertically or horizontally via the bubble center. This analysis shows the strong interface dynamics along the vertical axis; the preferential direction of pressure relaxation. The horizontal section evolution is less massive, but underlines the post collapse bubble fragmentation process. The directional fragmentation indicates a pressure field aligned at this moment along the Bessel central axis and relaxing perpendicularily. The symmetry is more or less persevered in the expansion phase. As the longitudinal expansion is less pronounced than the transverse one the points of maximal expansions are not occurring simultaneously in time. The longitudinal reverse movement commences earlier and the symmetry inverses and the decay favors now an elliptically transverse form. This symmetry inversion will induce a directional longitudinal jetting phenomenon perpendicular on the collapsing interfaces, sometimes accompanied by transverse streams.

**Figure 6:**
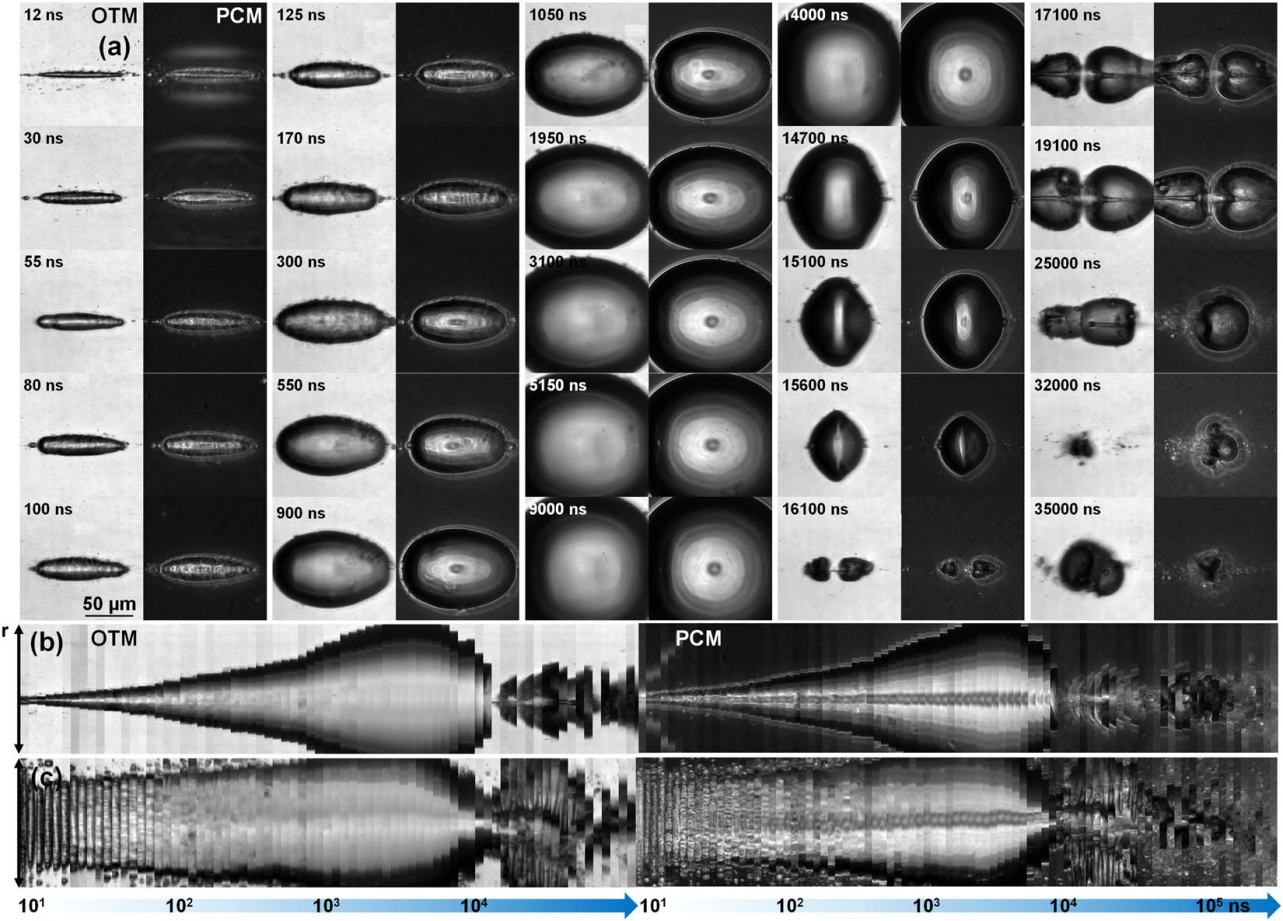
(**a**) Laser-induced cavitation dynamics in water and ethylene glycol mixture (70:30%) containing gold nanoparticles at a concentration of 1.3 × 10^12^ ł^−1^ for a tightly focused Bessel beam (*θ* = 16.5°) of 5 ps pulse duration and 6 *μ*J input energy. The dynamics is observed in OTM_*MSD*_ and PCM_*MSD*_ modes as indicated on the figure. Note that alternate columns represent OTM and PCM images respectively. (**b**) Dimensional dynamics of cavity evolution along the vertical axis. (**c**) Dimensional dynamics of cavity evolution along the horizontal longitudinal axis. A line section was vertically, respectively horizontally made through the bubble center at each delay time and the results were concatenated to form the image. The time was plotted on a logarithmic scale. The time labels are given with a tolerance of 10%.

**Figure 7:**
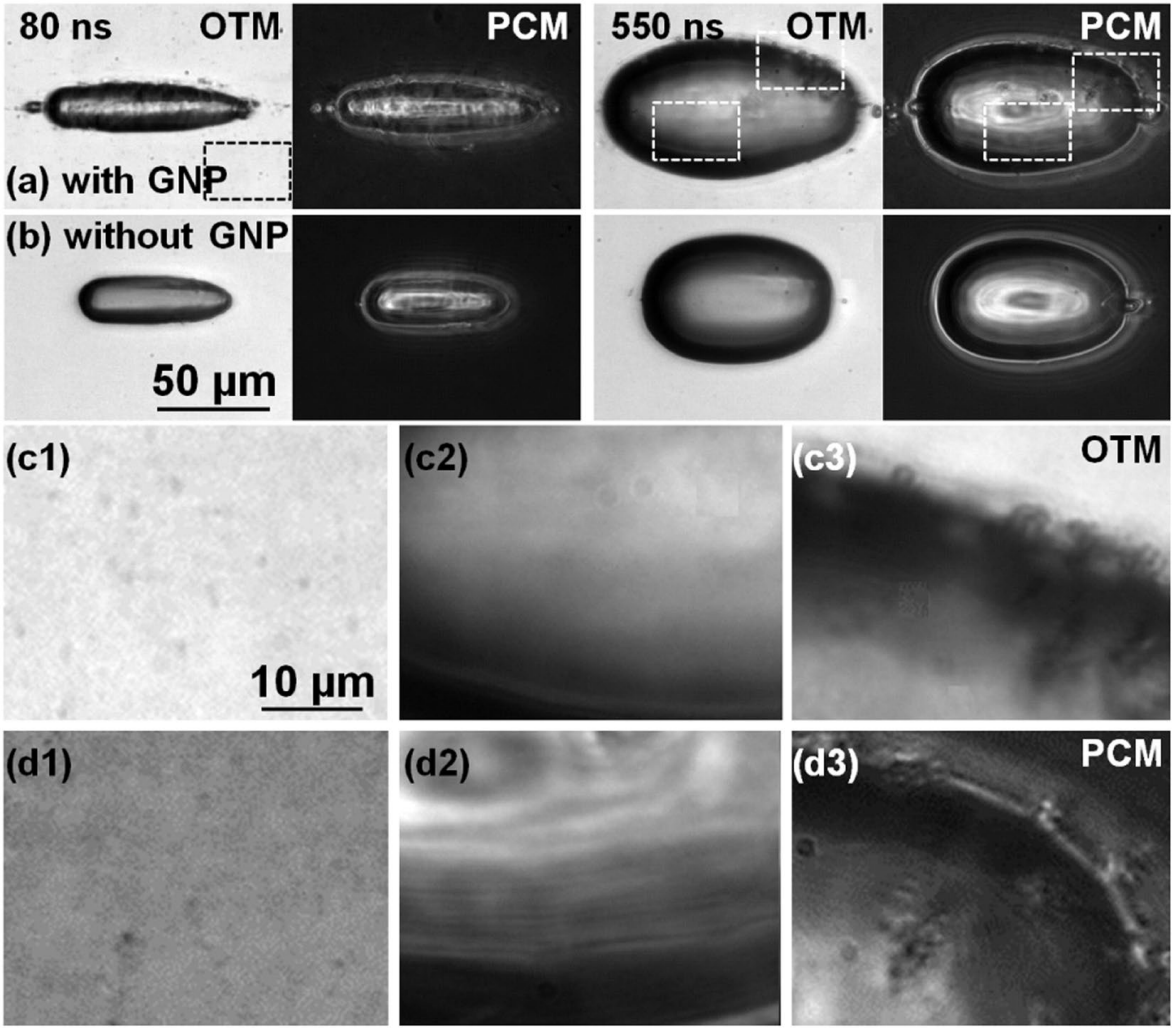
Particular time snapshots in the cavity evolution comparing the case involving the nanoparticles-containing-mixture of water and ethylene glycol (70:30%) (**a**) with the water case (**b**) in OTM_*MSD*_ and PCM_*MSD*_ modes (extracted from Figs 5 and 6). No GNPs are observed inside the bubble during the macroscopic expansion stage. For the nanoparticles-containing mixture, the evolving liquid-gas interface is hydrodynamically perturbed by the presence of nanoparticles, sweeping them away during expansion. (**c1**,**c2**,**c3**) Blow-up (4.5×) in OTM of the zones indicated in (**a**) for 80 ns-OTM, 550 ns-OTM, and 550 ns-OTM respectively. (**d1**,**d2**,**d3**) Same in PCM. The perturbed interface is visible.

A similar dynamics is followed in Fig. 6(a) in a water and ethylene glycol mixture (70:30%) containing gold nanoparticles at a concentration of 1.3 × 10^12^ ł^−1^ in comparable experimental conditions as those evoked in Fig. 5. The overall dynamics is similar to that observed in Fig. 5(a), albeit some optical disturbances in the illumination due to the scattering environment. This is confirmed by the dimensional analysis in Fig. 6(b,c). It is now of interest to observe the dynamics of the gas-liquid interface both in OTM and PCM mode. The interface is locally perturbed by instabilities caused by apparent obstacles which we associate with nanoparticles. An alternative explanation may involve the creation of micro-bubbles by local absorption at nanoparticle sites, though we have argued that the absorption section is not enough to induce local cavitation at these sites (not observed just below the macro-bubble threshold), and secondly, it is not clear how microbubble can resist in the bubble initiated in the whole excitation volume. We deduce that the nanoparticles are put in movement by the displacement of the interface, being trapped at the border by the superficial tension at small distortions. The moving interface creates a snow-plough effect. Figure 7 compares this situation for the two cases, with Fig. 7(a) showing the nanoparticles-containing liquid at two time moments (80 ns and 550 ns) and Fig. 7(b) showing the counterparts for the nanoparticle-free liquid. Blow-up of specific zones are given in Fig. 7(c1–c3, d1–d3) in OTM and PCM modes. They show the presence of GNPs in the initial liquid domain (Fig. 7(c1,d1)), the visually apparent absence of GNPs in the bubble at this delay time (Fig. 7(c2,d2), and the agglomeration at the interface (Fig. 7(c3,d3)) in form of disturbances. We conclude that, at the sensitivity of PCM and OTM, the particles are not able to be stored within the bubble during its expansion. They are driven by the expanding liquid, being trapped at the vapor-liquid interface.

## Conclusion

We have used an amplitude and phase sensitive microscopy technique capable of following bubble generation and relaxation on multiple timescales, ranging from picosecond to millisecond. Relying on ultrafast or low-coherence ns pulse illumination, the amplitude and phase information allows for resolving the excitation stage, expansion, and mechanical relaxation, with the visualisation of a kaleidoscope of hydrodynamical phenomena at the bubble collapse. The bubble onset overlaps with the electronic excitation. Its dynamics depends on the amount of laser energy, with the initial aspect ratio being determinant in the evolution. Generating bubbles in nanoparticles-containing liquids create a snow-plough effect that sweeps the nanoparticles at the gas liquid interface. This indicates that during the macroscopic cavity development, the nanoparticles were removed from the interaction region and dragged by the hydrodynamic movement. This illuminates the feasibility of preparing environments for nanoparticle excitation. It equally emphasizes the mechanisms at place during mechanical transfection using laser radiation. Manipulating laser-induced bubbles in liquids containing particles may find innovative applications in microfluidics, drug delivery, virus detection, and other biochip techniques. For instance, the combination of ultrafast particle accumulation within the bubble lifetime and the shape of bubble engineered in a cylindrical geometry by Bessel-Gauss laser beams would allow for a fast concentration and patterning of bio-particles (viruses) on biosensor surface arrays^48^ with enhanced sensitivity of their detection from biological media.
